# When Poverty Delays Care: The Silent Burden of Neglected Goiters in a World Where Basic Surgery Becomes a Luxury

**DOI:** 10.7759/cureus.100212

**Published:** 2025-12-27

**Authors:** Hamza Najout, Walid Atmani, Ilyass Masad, Mustapha Bensghir

**Affiliations:** 1 Department of Anesthesiology and Intensive Care Unit (ICU), Mohammed V Military Teaching Hospital, Mohammed V University, Rabat, MAR

**Keywords:** anesthesia, giant goiter, global health, low-resource setting, neglected surgery

## Abstract

Neglected thyroid disease remains a preventable cause of morbidity in low-resource regions, where poverty, limited surgical capacity, and geographical isolation delay access to essential care. In settings with reduced healthcare infrastructure, benign goiters may progress over decades, ultimately reaching massive sizes capable of compressing vital cervical structures. We report the case of a 62-year-old woman from an isolated mountainous hamlet in the High Zaïan foothills of Morocco who developed a giant anterior neck mass that had progressively enlarged for 25 years due to a complete lack of access to medical and surgical services. She presented with severe tracheal compression, dysphagia, and exertional dyspnea. Contrast-enhanced computed tomography revealed a giant multinodular goiter with marked rightward airway deviation and retrosternal extension. Because of the anticipated difficult airway, awake fiberoptic intubation under local anesthesia was performed, followed by total thyroidectomy, yielding a 1.8-kg multinodular specimen. The postoperative course was uneventful, and histopathology confirmed benign multinodular hyperplasia. This case illustrates how socioeconomic vulnerability and geographic isolation can transform a curable condition into a life-threatening deformity, underscoring the urgent global need to improve access to essential surgical care.

## Introduction

Thyroid disorders rank among the most widespread endocrine conditions globally, affecting hundreds of millions of individuals. While iodine deficiency and autoimmune thyroiditis remain common etiologies, the clinical trajectory of goiter varies substantially across socioeconomic contexts. In high-income settings, early diagnosis and elective thyroidectomy have nearly eliminated the complications associated with massive thyroid enlargement. In contrast, in low- and middle-income countries (LMICs), poverty, limited surgical capacity, health illiteracy, and geographical isolation frequently delay diagnosis and treatment. As a consequence, benign thyroid disease may progress unchecked for years, ultimately resulting in compressive symptoms, airway compromise, and disfiguring cervical deformities. Despite their benign origin, neglected goiters evolve into complex anesthetic and surgical challenges with potentially fatal implications [1,2].

Giant goiters are generally defined as long-standing thyroid enlargements causing marked cervical deformity, tracheal deviation, retrosternal extension, or significant compressive symptoms. Such presentations are associated with substantial clinical risks, including difficult airway management, dysphagia, positional dyspnea, and increased anesthetic complexity, particularly in settings where access to specialized care is delayed.

Beyond its clinical burden, the persistence of giant multinodular goiters in resource-limited settings reflects deeper structural inequities in global health systems. These cases embody the concept of “surgical poverty,” in which treatable diseases become debilitating because patients lack access to essential surgical care. Addressing such inequities requires investment in surgical workforce expansion, decentralized care pathways, community-based screening, and equitable financing models. In rural regions of Morocco, barriers such as shortages of trained surgical and anesthetic personnel, fragmented referral pathways, and infrastructural limitations continue to contribute to delayed presentation and treatment. This case from rural Morocco vividly illustrates how poverty and geographic isolation transform a readily treatable condition into a symbol of systemic failure - when poverty delays care, and when basic surgery becomes a luxury [3,4].

We present this case to illustrate how delayed access to essential surgical services can transform a typically benign thyroid condition into a surgical emergency, and to highlight the need for strengthened, decentralized surgical systems in resource-limited settings.

## Case presentation

A 62-year-old woman from a remote mountainous hamlet in the High Zaïan foothills, located in southern Khémisset Province (Rabat-Salé-Kénitra Region, Morocco), was referred to the Mohammed V Military Teaching Hospital in Rabat for evaluation of a massive anterior neck mass that had progressively enlarged over a 25-year period. Access to healthcare in her area was limited, with the nearest basic health facility located more than 40 kilometers away via unpaved mountain roads.

**Figure 1:**
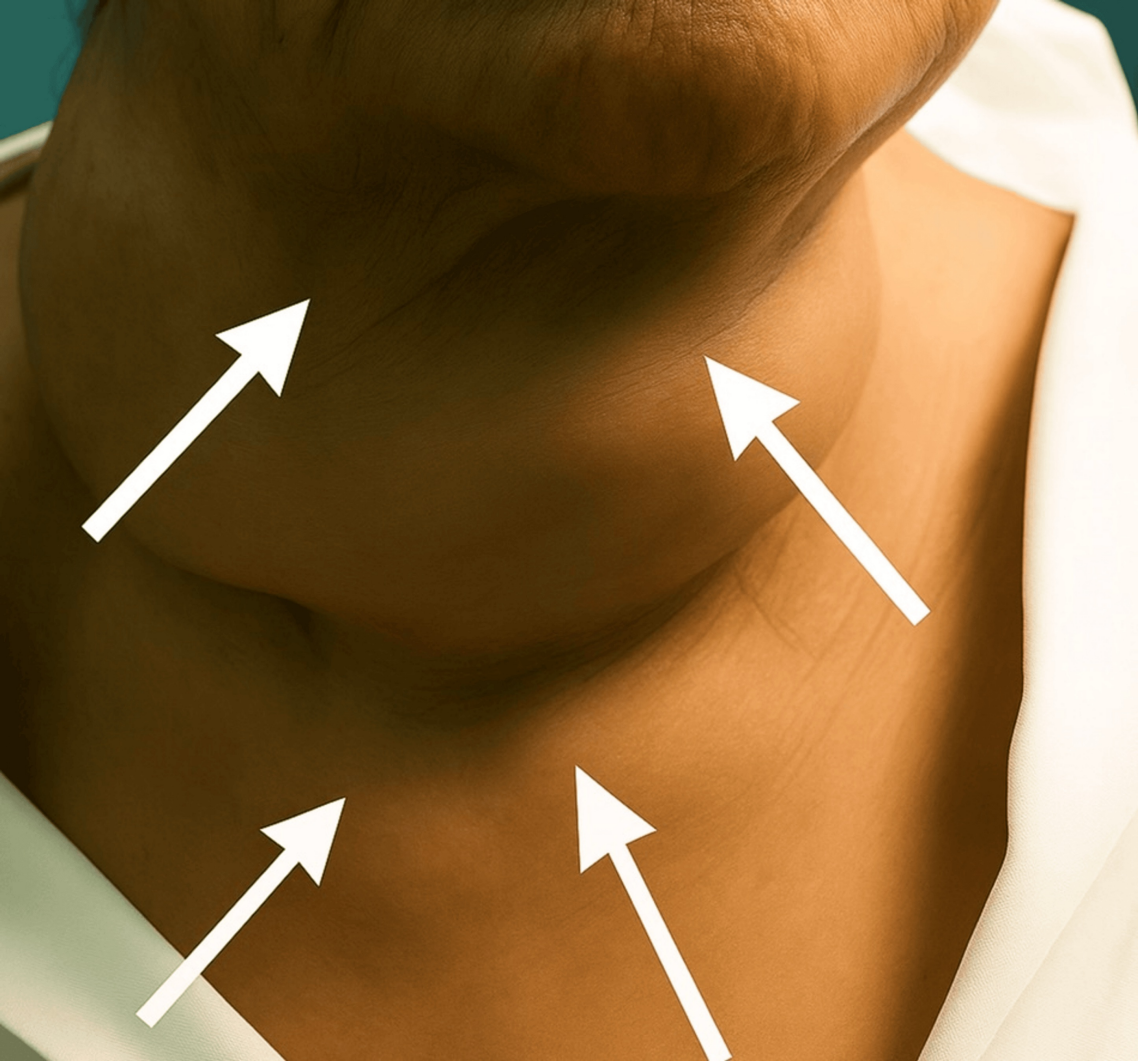
Clinical appearance of the anterior cervical mass. White arrows highlight the marked lateral and inferior contours of the giant multinodular goiter, demonstrating expansive cervical deformation and tracheal deviation.

Over the preceding decade, the patient developed progressive dysphagia, orthopnea, and exertional dyspnea, eventually requiring a semi-upright sleeping position. On admission, she was alert, hemodynamically stable, and appeared cachectic. Physical examination revealed a very large multinodular goiter occupying the entire anterior cervical region and extending below the sternal notch, associated with visible tracheal deviation and superficial venous congestion (Figure 1).

Laboratory evaluation showed normal thyroid function (thyroid-stimulating hormone 2.1 μIU/mL; free thyroxine 11.8 pmol/L). Cervical computed tomography demonstrated a giant multinodular goiter with retrosternal extension, marked tracheal compression, and rightward deviation of the airway. Given the anticipated difficulty in airway management, a multidisciplinary decision was made to perform awake fiberoptic intubation under local anesthesia, which was completed without complication.

**Figure 2:**
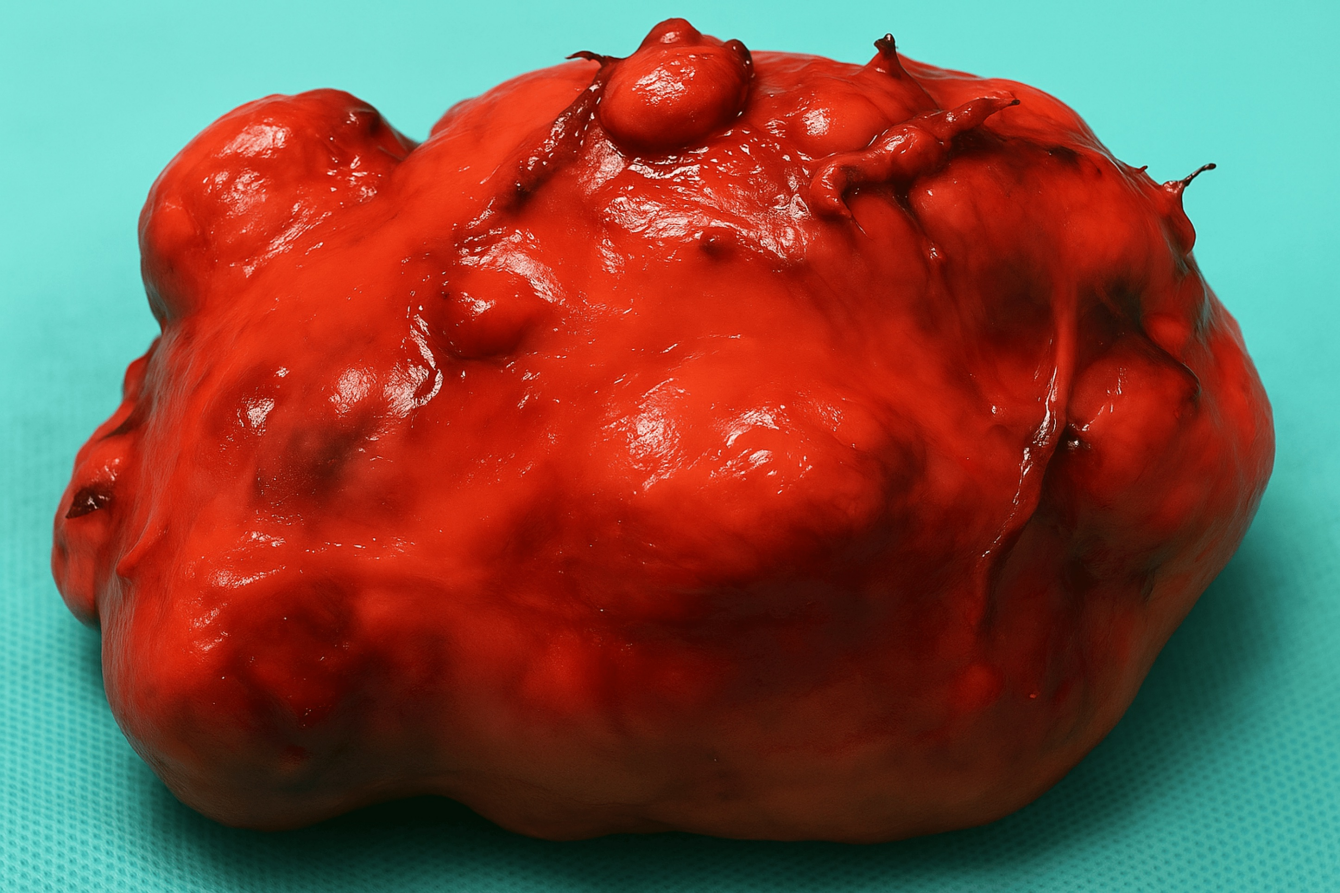
Macroscopic appearance of the surgical specimen. The resected multinodular goiter shows marked lobulation and irregular hypervascular surfaces characteristic of long-standing multinodular hyperplasia.

Total thyroidectomy was subsequently performed. Careful hemostasis was achieved, and both recurrent laryngeal nerves and parathyroid glands were meticulously identified and preserved. The excised thyroid gland measured 18 × 14 × 10 cm and weighed 1.8 kg (Figure 2).

The postoperative course was uneventful. The patient was extubated safely and experienced immediate improvement in respiratory comfort and swallowing. Serum calcium levels remained within normal limits. Levothyroxine therapy was initiated at a dose of 100 μg/day and subsequently adjusted based on serial thyroid function tests. The patient was discharged on postoperative day 5 with a structured endocrinologic follow-up plan, including clinical evaluation and thyroid function testing every six weeks during the first three months, followed by six-monthly assessments.

Follow-up was favorable in both the short and long term. At long-term follow-up, the patient remained clinically stable, with sustained resolution of compressive symptoms, no evidence of recurrence, and normal thyroid function under hormone replacement therapy. Support from the Social Service Division of the Royal Armed Forces facilitated transportation for follow-up visits and access to prescribed medication, ensuring continuity of postoperative care.

**Figure 3:**
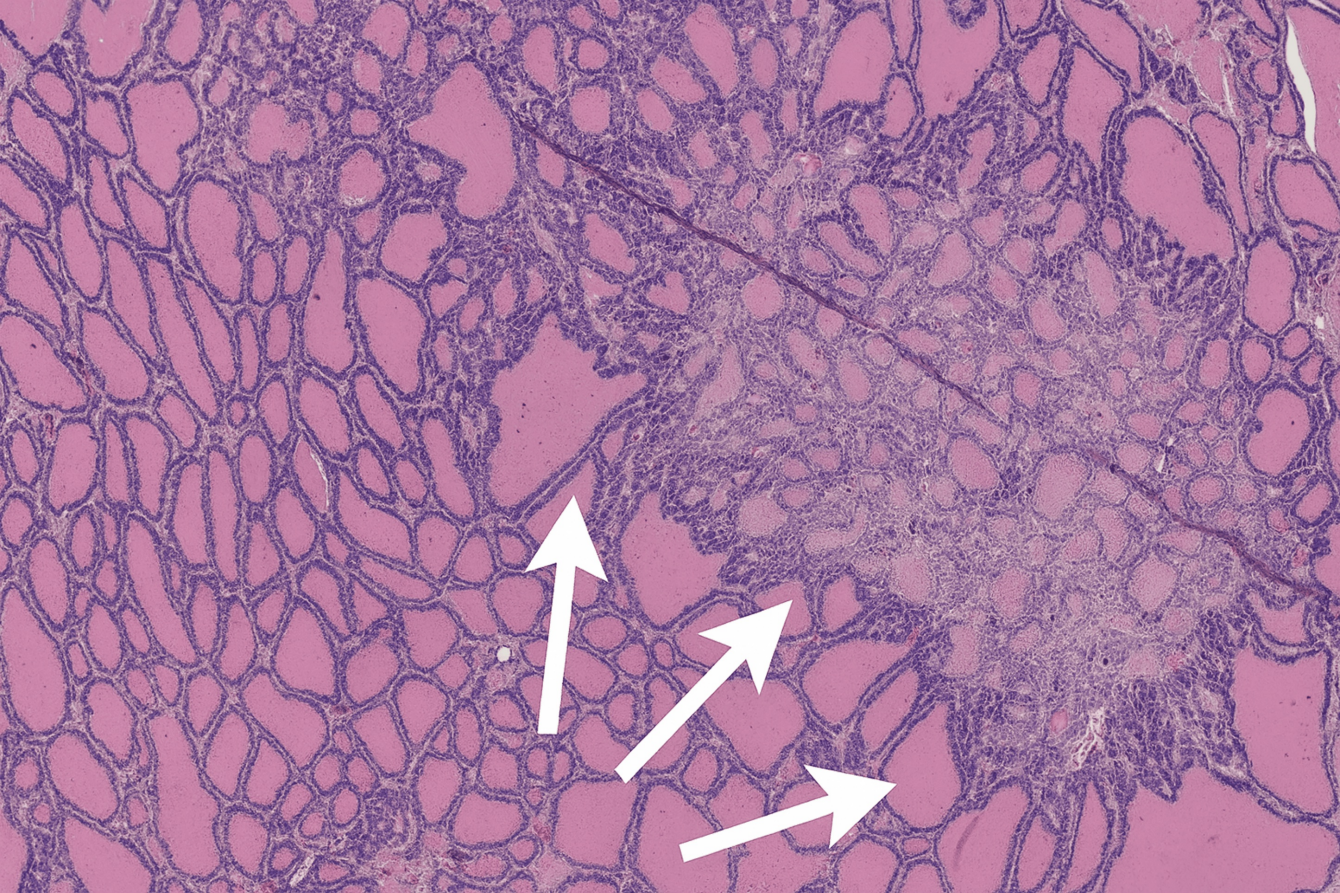
Histopathological features of benign multinodular hyperplasia (H&E stain). White arrows highlight irregular colloid-filled follicles separated by fibrous septa, consistent with multinodular hyperplasia.

Histopathological examination confirmed benign multinodular hyperplasia, characterized by variably sized colloid-filled follicles separated by thin fibrous septa and lined by cuboidal epithelium, without cytological atypia or evidence of malignancy (Figure 3).

This case was characterized by an exceptionally long disease duration, progressive airway and swallowing symptoms, positional dyspnea, and radiological evidence of tracheal deviation and retrosternal extension - features that should prompt early referral and careful perioperative planning.

## Discussion

This case represents an advanced manifestation of neglected thyroid disease rarely encountered in contemporary endocrine practice. Giant multinodular goiters exceeding 500 g have become uncommon in high-income countries, where early diagnosis and timely thyroidectomy prevent compressive complications [5,6]. Conversely, in LMICs, systemic barriers, including geographic isolation, inadequately equipped hospitals, workforce shortages, and financial constraints, allow benign goiters to progress over years or decades [7,8]. The patient’s 25-year evolution and extreme gland size (1.8 kg) positioned her at the severe end of the disease spectrum.

From an anesthetic standpoint, this case underscores the importance of anticipating airway obstruction in long-standing compressive goiters. Awake fiberoptic intubation remains the gold standard for anticipated difficult airways, particularly when tracheal narrowing or deviation is present, as endorsed by the American Society of Anesthesiologists (ASA) and the Difficult Airway Society (DAS) [9,10]. In this patient, multidisciplinary preparation ensured a safe and controlled airway management strategy.

Surgically, total thyroidectomy remains the treatment of choice for massive multinodular goiters to relieve compression and prevent recurrence [11]. However, such procedures are technically demanding due to hypervascularity, fibrosis, and distorted anatomy. Reported risks, including recurrent laryngeal nerve injury and hypocalcemia, can reach 10-11% in large goiters [12,13]. In this case, preservation of both recurrent laryngeal nerves and parathyroid glands allowed a complication-free postoperative course.

Beyond the clinical and technical aspects, this case highlights profound inequities in access to essential surgical care. The patient’s reflection - “surgery was something only city people could afford” - illustrates how socioeconomic and geographic barriers shape disease trajectories. The Lancet Commission on Global Surgery and the World Health Organization have emphasized that lack of access to timely, safe, and affordable surgical care affects billions worldwide and disproportionately impacts rural and marginalized populations [3,14]. Neglected surgical diseases, including giant goiters, therefore represent not only medical conditions but also indicators of structural health-system inequities.

The involvement of the Royal Armed Forces Social Service was decisive in ensuring postoperative continuity of care - an often-overlooked determinant of long-term outcomes in resource-limited settings. This integrated approach demonstrates how institutional support mechanisms can mitigate loss to follow-up and improve postoperative adherence for patients from remote regions.

## Conclusions

Neglected benign thyroid disease is a powerful reminder of the structural disparities shaping health outcomes globally. This case demonstrates how poverty, geographical isolation, and limited surgical capacity can transform a treatable condition into a disabling and life-threatening pathology. Ensuring timely access to essential surgery is a moral, clinical, and public health imperative. Strengthening health systems, decentralizing surgical expertise, and expanding outreach to remote areas represent key strategies to reduce preventable surgical morbidity in low-resource settings. Pragmatic measures such as mobile surgical outreach programs, targeted training of general surgeons in essential procedures, including thyroidectomy, and telemedicine-supported referral pathways may help prevent similar advanced presentations in underserved regions.
